# Anesthetic management in the lateral position in a patient with Parkinson’s disease who developed severe long-seated forward flexion with the face buried between the knees: a case report

**DOI:** 10.1186/s40981-025-00773-0

**Published:** 2025-02-14

**Authors:** Takayuki Morimoto, Masaaki Ono, Yayoi Harada, Taiga Ichinomiya, Ushio Higashijima, Tetsuya Hara

**Affiliations:** Department of Anesthesiology and Intensive Care Medicine, Nagasaki University Graduate School of Biomedical Sciences, 1-7-1 Sakamoto, Nagasaki, 852-8501 Japan

**Keywords:** Parkinson’s disease, Airway management, Lateral position, Video laryngoscope, Remimazolam

## Abstract

**Background:**

Camptocormia, a postural deformity seen in Parkinson’s disease (PD), complicates general anesthesia, especially airway management, owing to severe spinal flexion in advanced stages.

**Case presentation:**

We report the anesthetic management of a 76-year-old man with PD who developed severe long-seated forward flexion with the face buried between the knees, from camptocormia and multiple spinal surgeries. Removal of the exposed spinal implants was necessary, and general anesthesia was planned. Anesthesia was administered in the right lateral position from induction to awakening. Video laryngoscopy enabled successful intubation, and remimazolam with flumazenil ensured good recovery without complications.

**Conclusions:**

This case demonstrates the feasibility of managing the airway and administering anesthesia in the right lateral position in patients with PD with severe long-seated forward flexion. Video-laryngoscopy and remimazolam with flumazenil offer advantages in such cases, although further studies are required to validate their broader applications.

## Background

Camptocormia is a postural disorder involving abnormal forward flexion of the thoracolumbar spine that often develops with Parkinson’s disease (PD) progression [1]. Its prevalence spans 3–18%, and its response to pharmacological approaches for PD, such as levodopa, is unpredictable or disappointing [1]. Consequently, surgical intervention for posterior spinal fixation is required in severe cases. However, these surgeries have high reoperation rates owing to issues, including implant displacement or camptocormia progression [2, 3]. Additionally, multiple spinal surgeries may weaken the paravertebral muscles [4], potentially exacerbating lumbar curvature in conjunction with camptocormia. In advanced cases, general anesthesia can be challenging owing to difficulties with airway access. Although general anesthesia has been reported in camptocormia [5], its management in PD with severe long-seated forward flexion, as seen in this case, remains unexplored.

Here, we present a case of successful anesthetic management in a patient with PD with severe long-seated forward flexion caused by advanced camptocormia and multiple spinal surgeries.

### Case presentation

**Fig. 1:**
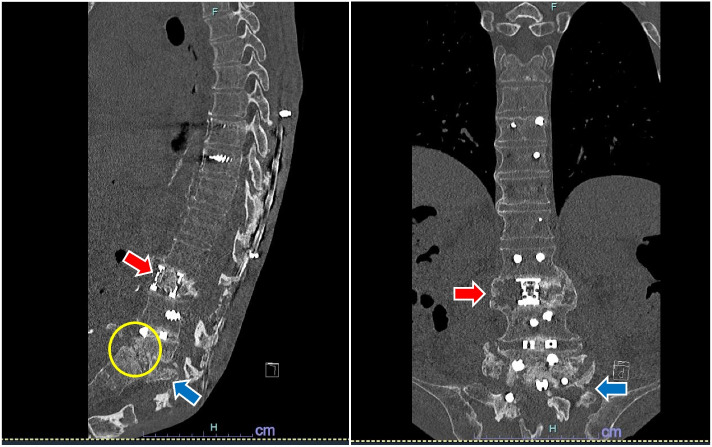
Degree of spinal deformity. Preoperative CT images (sagittal and coronal section). Postoperative posterior spinal fusion from the 9th thoracic vertebra to the 1st sacral vertebra. The 2nd lumbar vertebra (red arrow) and the 5th lumbar vertebra (blue arrow) are highly crushed, and the 4th lumbar vertebra and the 1st sacral vertebra form a false joint (yellow circle)

A 76-year-old man (height: 165 cm; weight: 47.9 kg) was diagnosed with PD 11 years prior and had progressively developed camptocormia. Three years prior, he underwent posterior spinal fixation from the 9th thoracic vertebra to the iliac bone; however, his camptocormia worsened, causing implant displacement. Consequently, implants from the 5th lumbar vertebra to the iliac bone were removed 2 years prior. Since then, progressive camptocormia, spinal deformity (Fig. 1), and paravertebral muscle weaking from multiple spinal surgeries led to a long-seated forward flexion in bed and a sacral pressure ulcer. Exposure of the spinal implants was observed beneath the ulcer pocket, leading to implant removal scheduling under general anesthesia.

**Fig. 2:**
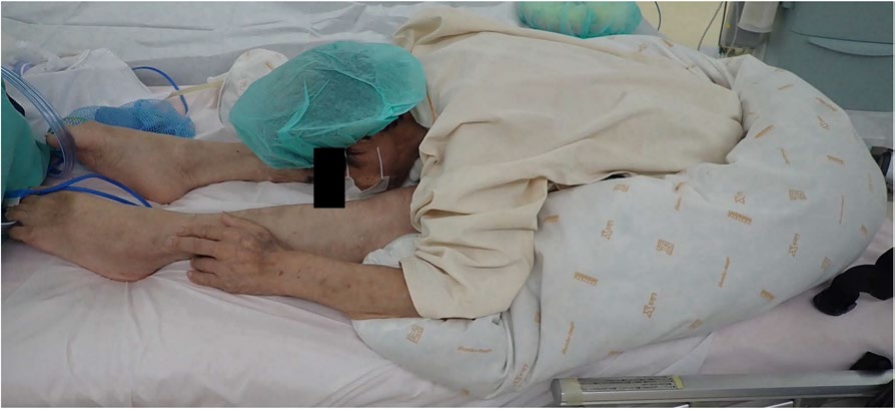
Severe long-seated forward flexion position. The patient’s face is buried between his knees

In the long-seated forward flexion position, he managed to eat with back support from towels; however, without support, his face was buried between his knees (Fig. 2). Preoperative respiratory function tests were not performed because of the patient’s position, but arterial blood gas analysis showed normal values (PaO_2_: 88 mmHg; PaCO_2_: 45 mmHg). Radiography revealed no evidence of tracheal deviation.

**Fig. 3:**
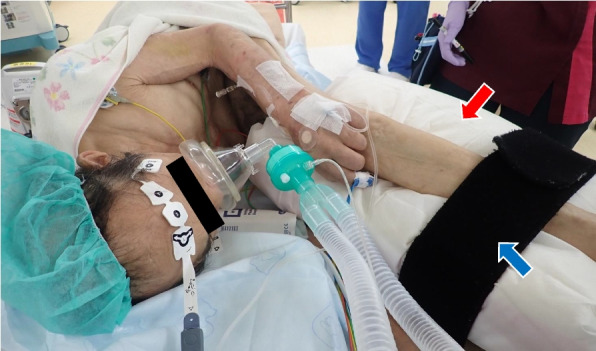
Space for airway management and positioning methods in the right lateral position. Sufficient space for airway management is observed in the patient while extending the lumbar region to the extent of no pain in the right lateral position. The patient’s legs were held by a limb band (blue arrow), and a pillow (red arrow) was placed between the legs to prevent nerve damage and pressure ulcers. The legs were not supported or pulled up by the staff to provide space

A multidisciplinary preoperative meeting was held to discuss positioning during anesthesia, surgery, and airway management. A simulation involving the patient, surgeon, nursing staff, and anesthesiologist was designed the day before surgery to evaluate possible positioning. Airway evaluation revealed favorable mouth opening and neck mobility, and the Mallampati classification was I. Airway management was deemed safe in either a supine position with legs spread or a right lateral position. Considering the patient’s discomfort in the supine position and the surgical requirement for a lateral position to access the sacral region, we decided to perform the entire procedure, including anesthesia induction, surgery, and awakening, in the right lateral position. This decision minimized the need for repositioning during surgery and allowed for adequate airway space by extending the lumbar region without causing significant discomfort to the patient. To prevent spinal deformity or implant exposure, lumber extension was stopped at pain onset. Legs were secured with limb bands, unsupported by staff, with pillows placed in between to prevent nerve damage and pressure ulcers (Fig. 3).

The patient’s PD was classified as Hoehn & Yahr stage V. His medication included a daily dose of 400 mg oral levodopa and six 4.5 mg transdermal dopamine agonist patches. On the surgery day, the patient received a morning levodopa dose; however, no intravenous levodopa was administered during the procedure, with the patches remaining in place.

**Fig. 4:**
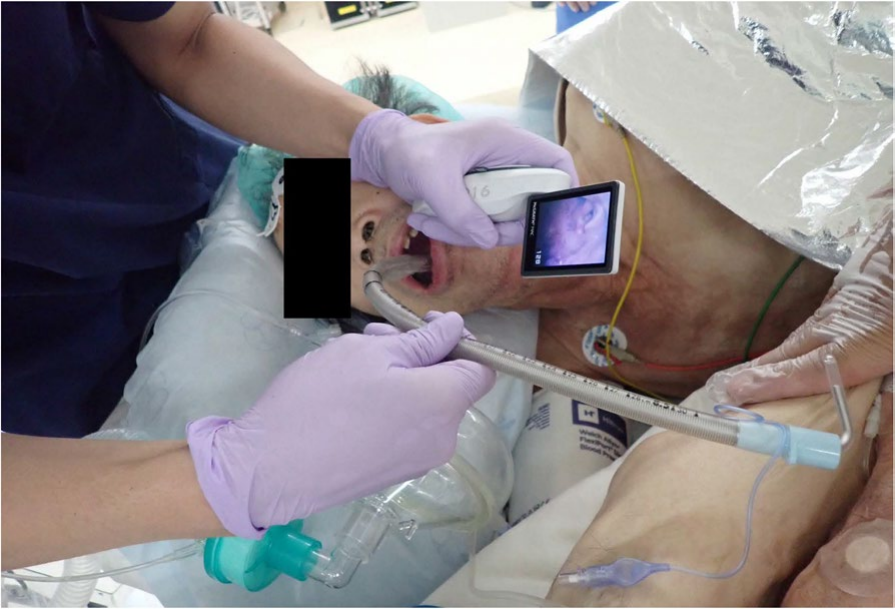
Intubation. Endotracheal intubation was achieved easily using the McGRATH™ MAC video laryngoscope, with a Cormack classification of I. The hand on the patient’s leg is the hand of the intubation assistant, and no force was applied to support or pull up the legs

Induction of general anesthesia was performed with 2 mg of remimazolam, 0.5 μg/kg/min of remifentanil, and 50 mg of rocuronium because airway management risks were deemed acceptable based on preoperative evaluation. In the right lateral position, mask ventilation was easy, and endotracheal intubation with an 8.5-mm internal diameter reinforced tube (Covidien Japan, Tokyo, Japan) was successfully achieved on the first attempt using the McGRATH™ MAC video laryngoscope (Medtronic, Minneapolis, MN, USA), with a Cormack classification of I (Fig. 4). Anesthesia was maintained at bispectral index monitor (Nihon-Kohden Corp., Tokyo, Japan) values between 40 and 60 using 0.5 mg/kg/h of remimazolam and 0.1–0.2 μg/kg/min of remifentanil. For postoperative analgesia, 100 μg of fentanyl was administered at the start of the surgery, and 750 mg of acetaminophen and 50 mg of buprenorphine were administered at wound closure. Before extubation, 0.5 mg of flumazenil was administered to facilitate awakening from anesthesia, and extubation was performed after confirming adequate spontaneous respiration. Despite the potential postoperative respiratory complications associated with PD, his respiratory status was stable, and the postoperative course was favorable. The surgical time was 94 min, and total anesthesia time was 207 min.

Subsequently, the pain from the pressure ulcer improved and, although passive, his lumbar range of motion increased, allowing him to sit in a wheelchair.

## Discussion

This is the first reported case of successful anesthesia management in a patient with PD that had progressed to severe long-seated forward flexion caused by camptocormia and multiple spinal surgeries.

In this case, anesthesia induction, maintenance, and awakening were performed with the patient in the right lateral position. Regarding airway management in the lateral position, previous reports have suggested that the visualization with direct laryngoscopy deteriorates when transitioning from the supine to the lateral position [6]. However, the use of video laryngoscopy in the lateral position has been reported to improve the Cormack classification and shorten the intubation time compared with direct laryngoscopy [7]. Some studies have indicated that video laryngoscopy can achieve a 100% intubation success rate in the lateral position, similar to that in the supine position [8], and that the success rate of intubation is equivalent between the right and left lateral positions [9]. Additionally, mask ventilation in the lateral position can prevent airway obstruction caused by gravity on the tongue and throat soft tissues. Consequently, the peak inspiratory and plateau pressures during mask ventilation are typically lower in the lateral position than in the supine position [8]. However, these studies excluded patients with predicted difficult airways. For patients with extreme lumbar curvature that limits neck mobility, unlike our case, awake airway management or alternative strategies may be necessary [10, 11]. Fiberscope intubation in the lateral position may be effective if performed by an experienced anesthesiologist [12]. Thus, when managing the airway in patients with extreme lumbar curvature in the lateral position, it is essential to first assess other airway management risks. If these risks are considered acceptable, mask ventilation and endotracheal intubation using video laryngoscopy can be performed without complications.

In our case, remimazolam was administered for anesthesia. Common perioperative PD complications include aspiration pneumonia because of muscle weakness and postoperative delirium [13]. In patients with PD, aspiration pneumonia prevalence has been reported to be 2.74% with a risk ratio of 3.3 [14], with the frequency of postoperative delirium 4.2% (odds ratio 1.88) [15]. Remimazolam is a sedative agent with a short context-sensitive half-life owing to its rapid metabolism into inactive metabolites and is equipped with the specific antagonist flumazenil [16]. Thus, these factors allow for an anticipated rapid recovery, which could be advantageous in mitigating the risks of perioperative complications in patients with PD. Some case reports have shown successful use of remimazolam in patients with muscular dystrophy or myopathy, avoiding postoperative respiratory complications [17, 18]. Other studies have reported comparable or lower rates of delirium with remimazolam compared to propofol [19, 20]. In this case, a good awakening was achieved without complications, such as aspiration pneumonia or delirium. Furthermore, although this case did not involve a predicted difficult airway, remimazolam use and its rapid reversibility with flumazenil are other key features that safeguard in the event of airway management failure [21]. This is the first report on remimazolam use in patients with PD, and further studies are warranted to clarify the benefits of remimazolam in this population.

In conclusion, in this case of sacral surgery in a patient with PD with advanced camptocormia and multiple spinal surgeries resulting in severe long-seated forward flexion, anesthesia was successfully managed in the right lateral position without problems. Even in cases of severe long-seated forward flexion, if airway risks are deemed acceptable, effective airway management can be achieved using a video laryngoscope in the right lateral position. Remimazolam with flumazenil may be useful in patients with PD because of its rapid recovery profile; however, further studies are required to validate these drugs.

## Data Availability

Not applicable.

## Abbreviations

PD: Parkinson’s disease
